# Strategies to Aid Successful Transition of Adolescents with Congenital Heart Disease: A Systematic Review

**DOI:** 10.3390/children10030423

**Published:** 2023-02-22

**Authors:** Pier Paolo Bassareo, Massimo Chessa, Giovanni Di Salvo, Kevin Patrick Walsh, Colin Joseph Mcmahon

**Affiliations:** 1 School of Medicine, University College of Dublin, D07 R2WY Dublin, Ireland; 2 Children’s Health at Crumlin, D12 N512 Dublin, Ireland; 3 National Adult Congenital Heart Disease Service, Mater Misericordiae University Hospital, D07 R2WY Dublin, Ireland; 4 Adult Congenital Heart Disease UNIT, Pediatric and Adult Congenital Heart Centre, IRCCS-Policlinico San Donato, San Donato Milanese, Vita Salute San Raffaele University, 20132 Milan, Italy; 5 Experimental Cardiology, Paediatric Research Institute (IRP), Division of Paediatric Cardiology, Department of Women’s and Children’s Health, University of Padua, 35128 Padua, Italy

**Keywords:** adult congenital heart disease, loss of follow-up, discontinuity in care, congenital heart disease, transition, epidemiology

## Abstract

The majority of patients born with congenital heart disease (CHD) need lifelong surveillance with serial clinical attendance and examinations. However, loss of follow-up (namely no documented follow-up for 3 years or more) is a recognised common problem since it is often related to remarkable worsening in the health of CHD patients with increased morbidity and mortality. Transitioning from paediatric to adult care has proven to be the most vulnerable point in the care of these subjects. As such, a systematic review was carried out to ask the following questions: What is the percentage of loss of follow-up worldwide? Are there regional fluctuations in the percentage? Is there a link between loss of follow-up and the complexity of CHD? What strategies should be employed to lower the risk of discontinuity in care? The most recent worldwide averaged loss of follow-up is 26.1%, with significant fluctuations across continents and countries. This percentage is even higher (31.9%) when one includes all untraceable patients, presuming that they are not having any cardiac follow-up. The highest discontinuity of care was reported in the USA and in patients with simple CHD. Planning the rules of transition seems to be one of the most reliable tools to minimise the number of CHD patients who are lost in transition. Recalling patients, with general practitioners who are crucial in readdressing half of the lost to follow-up CHD patients to adult CHD specialists, and a good relationship between paediatric cardiologists and the adult CHD team are two other valuable strategies in aiding successful transition.

## 1. Introduction

Congenital heart disease (CHD) represents the most commonly seen congenital defect, which frequently evolves from a life-threatening to a chronic lifespan condition. In fact, as a consequence of progress in the care of subjects with CHD, the survival rates have dramatically increased over the last few decades, with about 95% of children with CHD surviving into adulthood [1,2]. Consequently, the number of adult patients with CHD (ACHD) living today outweighs the new-borns and children affected by the same condition (70% vs. 30%) in the U.S. as well as in Europe. Likewise, survivors with complex CHD are on an upward trend, since approximately 10% of the ACHD patients are complex [3,4]. The International Society for Adult Congenital Heart Disease (ISACHD) judged that there are approximately 12–34 million ACHD patients worldwide, and 2.3 million of them are in the old continent [5,6]. 

ACHD patients may develop late cardiac complications and need serial examinations throughout their whole life [7,8]. In addition, premature death is common in ACHD patients, with cardiac insufficiency being usually responsible for death [9]. In Germany, only 22,000 ACHD subjects out of 330,000 adults with CHD are examined in ACHD accredited centres [10]. Over 200,000 ACHD patients are seen by general cardiologists [11]. This is not without risk, since CHD patients who are not examined by CHD specialists may receive inadequate care [12]. It has been widely shown that loss to specialist follow-up usually occurs at the moment of leaving a paediatric cardiology setting and entering an internal medicine or adult-oriented scenario [13]. During childhood, CHD patients are usually followed in paediatric cardiology centres, whilst ACHD programs are beginning to be run to provide cardiac follow-up throughout adulthood. During adolescence and with a certain degree of flexibility (12–18 years), CHD patients transition from paediatric to adult-care services [14]. 

Even though steady care is suggested for CHD subjects (for instance, the American Heart Association Guidelines point out that patients with CHD of moderate to high complexity should be examined at least every 2 years), unfortunately a large number of them present with care gaps or loss to follow-up [15], which means no documented cardiac follow-up for 3 years or more [16]. Adolescents with CHD are very prone to developing such care breaches due to their undergoing physiological, psychological, and social background. Transitioning implies switching care givers and, at times, hospitals. Discontinuation in care is harmful because it is related to a notably progressive worsening in the health of CHD patients [17]. In fact, loss of follow-up tripled the likelihood of needing urgent surgery or percutaneous procedure [18]. As such, with ageing, they are at high risk of cardiac complications such as the onset of heart failure, supraventricular and ventricular arrhythmias, pulmonary arterial hypertension, high blood pressure, clotting, infective endocarditis as well as of developing non-cardiac pathologies [7]. Lapse in the transition pathway may cause a delayed acknowledgement of these ongoing cardiac and non-cardiac issues, thus complicating the management of these patients [19].

Planning the rules of transition has a likely key role in preventing these risks [20]. Informing CHD individuals and their caregivers on the reasons for undergoing follow-up is crucial as they should become responsible for themselves [21]. In the absence of a formal transition program, adolescents and young adults affected by CHD often do not know much about their condition and related outcomes [22,23]. On the other hand, when a formal program to manage transition is lacking, patients expose themselves to the possibility of a delay in the transfer of care or receiving inadequate care, with consequential unpleasant emotional and financial issues for them, their families, and the health care system in general [24].

The number of CHD patients experiencing discontinuity in care varies a lot across studies, countries, and disease complexities. Again, it is still debatable as to what extent transition programs are able to prevent the loss of follow-up from occurring [25]. 

This review asks the questions (a) What is the percentage of loss of follow-up world- wide? (b) Are there any regional fluctuations? (c) Is there a link between the loss of follow-up and the severity of the heart disease? and (d) What strategies should be employed to lower the risk of discontinuity in care?

## 2. Materials and Methods

### 2.1. Search Strategy 

A literature review was conducted investigating the electronic databases PubMed, Scopus, and Web of Science from their establishment up to 31 December 2022 (PRISMA statement see Supplementary Materials). The MeSH (Medical Subject Headings) terms “transition”, “congenital heart disease”, “adolescence” and/or adolescents”, and “loss of follow-up” were searched. Papers in languages other than English were excluded. 

### 2.2. Study Selection 

The authors separately looked into the selected abstracts and evaluated whether they were eligible. Full-texts were checked when all of the reviewers of the abstracts felt that the latter might match the inclusion criteria. 

### 2.3. Data Extraction 

Information extracted from the selected manuscripts were best practices, guidelines, expert opinions, literature reviews, transition programs, and surveys evaluating the transition for adolescents affected by CHD.

**Figure 1:**
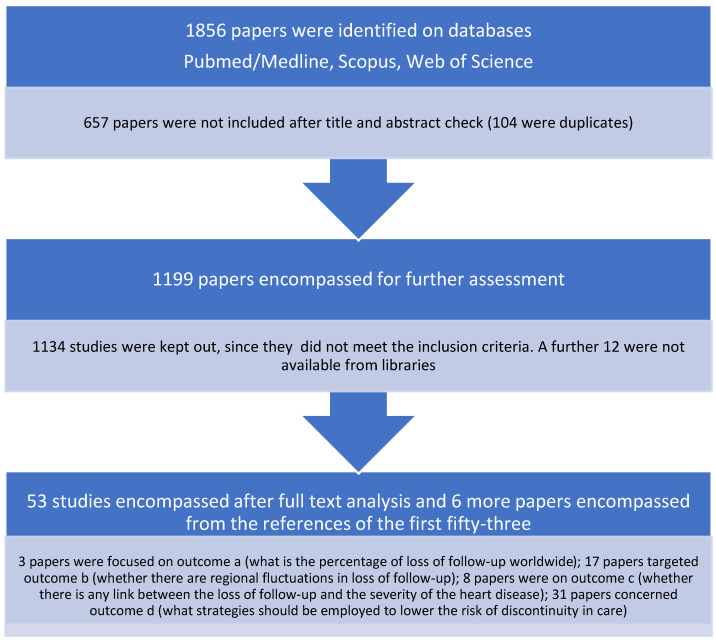
Flow diagram of the study selection process.

A total of 1856 potentially eligible studies were found, of which 657 (104 duplicates) were removed after the screening of their title and abstract. Thus, 1199 studies were encompassed with a view of full-text assessment on the basis of title and abstract. A total of 1134 were removed as they did not meet the inclusion criteria, and 12 were not available from online libraries. As such, 53 studies met the inclusion criteria, and six additional papers were encompassed after checking the references of the first 53. A flowchart of the study selection process is shown in Figure 1.

Three manuscripts focused on question (a), 17 articles had question (b) as a target, 8 articles analysed question (c), and 31 articles on question (d) were found.

## 3. Results

### 3.1. Proportion of Loss of Follow-Up around the World

As above stated, the risk of loss of follow-up is likely to be the foremost weakness of transition during adolescence. Discontinuation may begin at an early age, even for CHD patients with defects of high complexity, with percentages showing ups and downs worldwide, but with an average value of 26.1%, as shown in the most recent meta-analysis released in the field. The percentage is even higher (31.9%) when including all untraceable patients and supposing that they did not have any follow-up. The true discontinuity value is expected to be within this range [26]. When specifically focusing on continents and regions, discontinuity in care was 34% in the USA, 25.7% in Canada, and 6.5% in Europe [26]. However, it is noteworthy to highlight that the 17 studies that had been enrolled in the meta-analysis were not homogeneous, and Europe was underrepresented with only two studies carried out in Sweden and Belgium. Again, there were two conference abstracts that had not been considered for meta-analysis since they were not published as extensive articles [26]. Another possible explanation of such a difference is the fact that many European countries guarantee universal access to health care, while in the USA, access is easier for those who are wealthy [27]. In addition, the distance from the place of living and the referral ACHD centre might at least in part explain the discrepancy between the USA and Europe regarding the loss of follow-up during transition. For example, in the USA, about 45% of the population lives >1 h to an ACHD centre, and 5.4% lives >4 h away from one [28]. 

### 3.2. Regional Fluctuations in Loss of Follow-Up 

Discontinuation in the care of CHD shows ups and downs around the world. Regarding Europe, even on a small island such as Malta, with easy access to the health care system, the loss to follow-up was 21%. Nevertheless, following being recalled from hospitals, more than 80% of the initially lost patients accepted being examined and had their regular follow-up [29]. In Belgium, discontinuity in follow-up was quite low, with a reassuring 7.3%. Multivariate statistics showed that male gender and no previous cardiac surgery were correlated to interruption in care [30]. Furthermore, in the Netherlands, transitioning from paediatric to adult care was linked to loss of follow-up of approx. 18% of the sample. As already noted in Belgium, male gender, a non-severe CHD, but also a past medical history of follow-up in a local hospital setting were correlated with discontinuity in care. In contrast, patients frequently admitted to hospital, with prior invasive interventions, having complex CHD, taking medical therapy, having received transition education, and frequently examined since early childhood had an increased likelihood to transition to adult cardiac care smoothly [16,31]. However, another different estimation of loss of follow-up in the Netherlands has also been released. A national registry and DNA bank of ACHD, named CONCOR (CONgenital COR vitia), was developed by the praiseworthy initiative of the Interuniversity Cardiology Institute of The Netherlands and the Netherlands Heart Foundation at the beginning of the new millennium. The CONCOR registry showed that 17,000 ACHD subjects were receiving regular cardiac care in Holland, whereas another 8000 (47%) patients did not for some reason [32,33]. In a Swedish multicentre study carried out in seven university hospitals, 10.3% of the sample were lost to follow-up or untraceable. Hospital related factors such as a high number of patients at outpatient clinics, but no medical staffing resources or patient-related features (such as gender or disease complexity) were linked with continuity in care [34]. In a single ACHD centre survey carried out in the UK, nonattendance in all ACHD patients was 10% [35]. Even when considering a single defect of moderate complexity such as Tetralogy of Fallot in the UK, 24% of living patients appeared not to be followed-up in specialist centres, although a few of them were having follow-up in general cardiology clinics. The average length of loss to follow-up was 22 years. A significant number of patients had been lost before ACHD units were formally instituted in the UK. Half of the late deaths were registered in Tetralogy of Fallot subjects not having any specialist surveillance [13].

Concerning the USA, in a single centre study (Columbus, OH, Canada), the detected loss of follow-up was 37.6%, thus highlighting a suboptimal maintenance of care, even in a single setting [16]. The highest prevalence of loss of follow-up in America was found by Yeung and Coll (62.7%) in patients of moderate or high complexity [18]. A similar prevalence was also shown in the study by Vaikunth and Coll (61.2%), in spite of a formal transition program being in place [36]. Both studies concluded that such a high prevalence tended to lead to poor outcomes [18,36]. On the other hand, the lowest prevalence was at the Boston Children’s Hospital (8.7%), which has an ACHD care clinic on-site, in spite of managing patients of comparable complexity [37]. This may highlight the importance of having an ACHD clinic structurally connected to the paediatric cardiology clinic.

The highest outlined percentage of loss of follow-up was in Canada, with an astonishing 53% in the research by Reid and Coll. They carried out their study at the largest paediatric cardiac centre in the country (Hospital for Sick Children, Toronto, ON, Canada). The prevalence halved (24%) after recalling the patients [38]. A similar percentage (49.2%) was in the study by Mackie AS et al. [39]. The lowest prevalence (3.6%) was that found by Mondal TK and Coll in a single university centre study offering paediatric and ACHD clinics in the same site. This is probably the explanation for such an astonishing difference in loss to follow-up [40].

**Figure 2:**
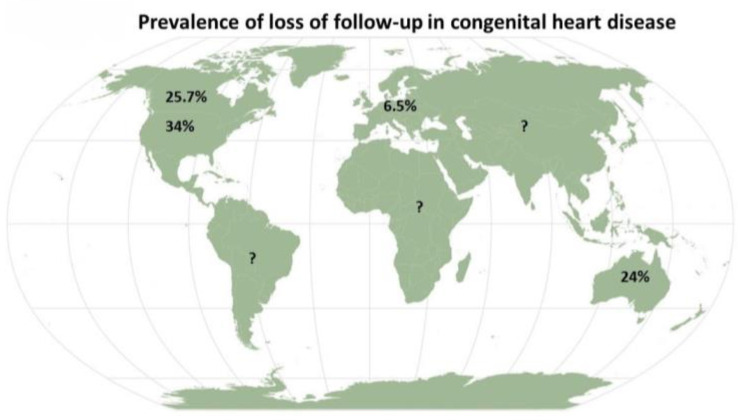
Continental prevalence of loss of follow-up.

Data from Australia are limited to the form of an abstract showing a loss of follow-up of 24% in patients with CHD of moderate and high complexity, with a slight female prevalence [41]. No data are available from South America, Africa, and Asia so far (Figure 2).

### 3.3. Relationship between Disease Complexity and Discontinuation of Care

As per the AHA/ACC classification, CHD are subdivided into three classes on the basis of the degree of complexity [8] (see Table 1).

It is generally accepted that those affected by complex CHD are more likely to discontinue their follow-up [30,31,42]. The same is true in patients who required device implantation [36]. However, when pooling all data by multivariate analysis, in spite of the loss of follow-up being prevalent in ACHD patients with simple defects, the difference proved not to be statistically significant [26].

The results of the meta-analysis are very important, since single centre or regional studies keep sharing the idea that patients with less severe CHD have an increased likelihood to be lost in follow-up. For example, this was outlined by a recent American study carried out in North Carolina (e.g., an American state with the highest percentage of gaps in ACHD patients care compared to the others) [43]. However, some significant study limitations should be highlighted, which may have influenced the findings. First, the definition of loss of follow-up was a 2-year gap in care rather than the usual 3 [16], and second, the fact that over 25% of subjects affected by a severe CHD did not have any follow-up by 2 years [43].

### 3.4. Strategies to Lower the Loss to Follow-Up

Reducing the number of CHD patients who are lost in their care is extremely important, since they have a marked tendency to turn up in the Emergency Department due to a sudden worsening in their clinical state. About 20% of those urgently admitted pass away or require heart transplantation by 3 years from the initial admission [44]. This was supported by Mylotte et al., who confirmed that the patients who do not receive specialist follow-up are more likely to die [45].

Social determinants of health such as poverty, lack of private insurance, difficulties in housing, low level of parental education, being an immigrant, belonging to ethnic minorities, shortage of food supply, and transport barriers are linked to a number of adverse outcomes, missed examinations, with loss to follow-up included [46]. As such, social determinants of health evaluation and referral to appropriate social services may lower the discontinuation in care [46]. This is the concept of “syndemic” (e.g., the theory stating that issues come up from the complex interaction between the spreading of an illness and social and/or environmental and/or economic circumstances that in turn negatively affect the illness itself) [47]. These individuals require specific attention to prevent discontinuity in their care.

Overbooked paediatric cardiology clinics with a lack of time to address the multiple issues concerning transition is another possible explanation of lapse in the continuity of care that should be amended. This often implies transferring patients when they are still too immature or also after too few paediatric outpatient examinations in the pretransfer period. A survey carried out among CHD caregivers reported that only 31% thought that their adolescent patients had been appropriately prepared for transition [48]. For these subjects, a single chat about transition at the time of the last paediatric examination might not be enough. Not only is an earlier discussion needed, but other tools such as setting up specific transition clinics are also needed.

Likewise, adolescent patients who are at risk of or inappropriate behaviours such as drug abuse may necessitate more aggressive programs to make significant progress in their way of acting, which may lead to successful transfer [38].

The involvement of a nurse in the transition clinic (multidisciplinary team), with the aim of spending time in helping CHD adolescents develop a comprehensive understanding of their condition, self-care management, and the skills that are needed to take part in adult life is another reliable strategy to prevent any lapse in care [49].

General practitioners are crucial in returning half of the lost to follow-up subjects to specialised supervision. Intensifying cooperation with them may be a way to further lower lapses in care [50].

A structured transition programme ensuring a planned and coordinated transfer has been suggested to be the most important tool to prevent any discontinuity in care from occurring. Unfortunately, in the clinical setting, even when some resources for transition are available, they are often represented by non-tailored educational leaflets about CHD and do not address the preparation to transition appropriately during this high-risk process [51]. Conversely, specific programs should be developed for an appropriate approach to transition that enrols specialised health care personnel pushing the regular reinforcement of concepts and running transition periodic assessments with the involvement of patients as well as their families [52]. For a long time, it was widely accepted that loss to follow-up or gap in care starts exactly at the moment of transferring follow-up from a paediatric to an adult scenario. Conversely, more recently, it has been perceived that CHD patients who are lost in transition are more numerous than that previously supposed and that the loss of follow-up starts when the patients are children, adolescents, and teenagers. This implies that to prevent the loss of follow-up from occurring, a timely transition programme is mandatory. This should start before the significant loss of follow-up begins (e.g., at early adolescence). Unfortunately, the current number of health care personnel is not sufficient to look after the great extent of transition patients in society. Therefore, it will be necessary to set up more specific ACHD programmes, to teach more ACHD cardiologists, to increase the number of transition programmes, and to hire transition nurse specialists on staff [53]. Even the employment of administrative staff and managers who are totally dedicated to transition may be another way to keep transition patients in care [34].

In an ideal tertiary care centre, joined facilities for paediatric and adult cardiology should be offered. Proposing joint appointments with paediatric cardiologists and ACHD specialists for a formal handover of care should reduce the risk of losing the care of CHD patients [54]. If this is impossible, having a strong conjunct transition programme is crucial. The Society of Adolescent Medicine states that transition is “the purposeful, planned process that addresses the medical, psychosocial and educational/vocational needs of adolescents and young adults with chronic physical and medical conditions as they move from child-centred to adult-oriented healthcare systems” [55]. Preparation for being independent is an ongoing and lengthy activity that usually begins at the ages of 12–14 and is completed by the age of 18 years [56].

Interestingly, a study highlighted that the main cause for loss to follow-up is represented by the fact that the CHD patients were told that there was no need for any further examination. This occurred in about a third of the samples [18]. Lack of or poor communication represents one of the major challenges capable of affecting the quality of transition in a number of scenarios, as previously tested. For example, inadequate communication at the time of discharge proved to cause issues with drug orders and incorrect information concerning the patients’ current health status. To obtain the situation sorted, repeated clarifications were needed by phone, thus triggering a delay in care, increased stress in health care staff, and frustration in the patients and their family members, which contributed to negative health care images and increased risk of re-hospitalisation [57].

Some patients have a very poor knowledge of their CHD and related needs, which may explain, at least in part, why they are lost to follow-up. When specifically giving insights into the problem, among the male patients, the highest knowledge deficit was about medical problems whilst among the females, the highest deficit concerned lifestyle issues. Lack of knowledge in pregnancy-related risk, probability of disease recurrence, and family planning was alarming as it involved approximately half of the samples. Teenagers and young adults suffering from simple CHD had the lowest knowledge deficit [58]. The latter tends to increase with the patients’ age [59]. The detected discrepancies regarding health status knowledge between the two genders might be related too different interests, parental worries or beliefs, or caregiver counselling bias. Those with simple CHD may wrongly think that their heart defect is definitely “treated” or “healed” [58]. It is noteworthy to highlight that previous research has suggested that poor knowledge triggers increased anxiety, an improper approach to health care, and uncertainty in decision-making [60].

CHD represents a significant stressor triggering psychological disturbances since birth. Prolonged hospital stay as a neonate as well as social limitations and bullying as a child or adolescent may cause anxiety and depression, which can even be exacerbated when transitioning from paediatric to adult services. In this respect, psychologists may help both patients and their parents and reduce the loss of follow-up [61].

As previously mentioned, there is a considerable number of international transition projects such as formal education programmes, nurse-led actions aimed at ameliorating ACHD patients’ knowledge, or the establishment of a mobile app to reach young patients. All of them show promising results and are focused on trying to set up a smooth and properly working transition process [62]. Specifically regarding the last approach, it is widely known that adolescents are interested in Internet and mobile-based programs [63]. This is particularly evident in young people belonging to ethnic minorities. For instance, among Latinos aged 18–29 years, two thirds have a smartphone and one third a computer [64]. Again, 98% of African-Americans in the same age range have either a broadband connection or a mobile phone [65]. Recent research has found that African-American teenagers are more likely to use social media platforms and messaging apps than their White counterparts [66]. Based on these premises, with the help of an advisory council of adolescents, a mobile app was set up. Four points were taken into account in developing it, namely:

1. Creating a tailored app with a few things such as a specific diagram for each patient, a transition checklist, a way to monitor progress throughout the list, a past medical history background, notifications of appointments, and therapeutic changes;
2. Establishing education for patients with video clips for each specific disease with links to learning topics such as the way to recognise an emergency;
3. Promoting mentorship from peers and ACHD patients suffering from the same condition and enabling connection with each other by means of chats;
4. Creating blog spaces and forums for patients to share their experience, ask medical questions, and make comments about the other patients’ observations [63].

Since CHD is the most commonly seen congenital abnormality, and, as previously mentioned, living ACHD patients outnumber patients surviving childhood, giving teenagers the power of properly transitioning care is extremely important. It has been previously outlined that personally managing chronic diseases requires five main skills: problem solving, decision making, resource utilisation, patient–doctor bonding, and taking action [67]. The mobile app that was created is able to provide assistance in addressing the first two points (e.g., problem solving and decision making) by sharing CHD details and lifestyle suggestions and developing portable tools, a medical synopsis, and specific diagrams for each CHD included. These can be used by adolescents to convey important information about their disease [63].

In Quebec (Canada), due to the high number of ACHD patients missing appropriate care, a national media campaign called “Wanted! 8000 Heart Patients” was launched by means of billboards displayed all over the country. The publicity campaign generated close awareness from ACHD individuals and the media. After a short time, many articles were released in newspapers and magazines and attention was generated via national radio and TV. As such, 800 more ACHD patients were referred to appropriate care. The successful campaign represents an example of a commendable effort to raise awareness among people and regain those ACHD individuals who were not on any follow-up [32].

Another way to retrieve ACHD patients who are experiencing a lapse in their care might be that suggested by Diallo et al. [68]. Since lapses in specialty care are sometimes represented by the lack of patient documentation in electronic health record systems, a risk scoring system was released. The latter was simple and based on age, gender, and electrocardiographic parameters. This demonstrated 96.4% sensitivity and 80% specificity at a threshold score of 10 in identifying ACHD patients with moderate to severe complexity [68].

In the recent document released by the AHA on transition for CHD individuals, three bullet points were identified as the most important in the whole process of the transition of care, namely:

1. Features that are specific to ACHD populations including the influence of social factors determining health, well-being, and neurological status;
2. The additional costs for the health care system and potential health complications that are related to inadequate transition to ACHD care including the increased access to the Emergency Department;
3. The need to set up tailored transition programmes addressing and implementing the participation of CHD adolescents in face-to-face and online appointments with care givers, adopting a family-centred approach [69].

Even in the most recent (and so far only) meta-analysis released in the field, the loss to follow-up number was clearly lower in those subjects formally involved in a transition program in comparison to those who were not. Nonetheless, this differentiation was not statistically significant. This is likely to be related to the fact that only three studies comparing CHD patients with or without the provision of transitional supervision were considered. Further research on the transition in ACHD patients will likely shed light on this interesting point [21,70,71,72].

## 4. Discussion

The process of transition is really important, since the number of teenagers who are in the transition age is estimated to account about 15–20% of the overall population with CHD [73]. This number might be even higher when considering immigrants and non-residents, whose numbers are difficult to evaluate and vary across countries [69]. In the past, the acronym GUCH (Grown-up Congenital Heart patients) was coined to term these subjects [74]. While those with a simple CHD can be seen in a general cardiology setting, patients with a moderate to high complexity in terms of anatomy and/or cardiac function should be transitioned to specialised centres for the care of CHD [69].

However, as mentioned, in a not negligible percentage of CHD individuals, discontinuity of care represents an issue. The reported numbers are unacceptably high, as they are significantly related to a high risk of health deterioration, the need for a heart transplant, and death. Some strategies involving multiple stakeholders seem to be promising in lowering the lapse in care, but further prospective research will be needed to confirm this. For instance, having a CHD-specific transition program will save families and adolescents the stress of figuring out the process themselves. Professional advice and progressive health care transition from early adolescence, in addition to obtaining information and encouragement from patient family organisations, will undoubtedly have a positive effect on the outcomes for those suffering from CHD and their parents, including reducing the loss of follow-up [69].

Health care transition is made up of two interconnected factors: the transfer of medical follow-up from paediatric to adult health care professionals, and the transfer of medical responsibility from parents to young patients [69]. A good transition should reach the aims of (1) improving the patients’ knowledge on their CHD; (2) pushing the patients’ self-management and self-advocacy with a view to achieving independence; (3) learning how to manage a medical condition that can be complex; and (4) promoting integration into adult-centred care for CHD and non-cardiac comorbidities [75].

An important concept that is linked with transition is the so called “quality of life” (QoL). In fact, well-being is another goal of transition. The ultimate goal of a smooth transition programme is increasing the QoL of young patients, along with their life expectancy. QoL has the potential to be implemented through continuous health care. The latter minimises admission to hospitals and promotes physical and psychosocial well-being and QoL. A recently published review showed that appropriate transition interventions lead to psychosocial benefits such as improving QoL, CHD self-management, and the state of being fully prepared for transition. Additionally, the likelihood of an appropriate transition increases when CHD patients judge life positively and feel good [76].

Many tools are available to measure the adolescents’ transition readiness with time [77], of which the Transition Readiness Assessment Questionnaire (TRAQ) V4.0 is one. It comprises five topics subdivided into 29 subtopics to cover all areas of transition in adolescents with CHD [39,78]. Nevertheless, the TRAQ has its own limitations, for example, in measuring self-advocacy [79]. The TRANSITION-Q is another questionnaire with 14 items to be tested to verify the efficacy of the proposed structured transitional intervention [80,81]. The higher the score in the questionnaires, the higher the likelihood of a smooth transition. There are also other less diffuse tools to measure CHD knowledge (e.g., the Leuven Knowledge Questionnaire; the MyHeart scale) or competence in understanding your own health status (REALM Short Form) that can be used in selected cases [82,83,84].

Current data on the loss of follow-up are quite complete regarding North America, whereas Europe is under-represented with only two studies from Sweden and Belgium, as included in the only meta-analysis in the field. These are not necessarily representative of Europe. As an obvious consequence, more research in Europe will be required to check whether the apparently reduced loss to follow-up is also confirmed in other European regions, especially in the south of the continent. Furthermore, robust research in the field should be carried out in South America, Asia, and Africa, since so far, data are missing for all. Specific funding for such studies in low-income regions must be allocated.

**Figure 3:**
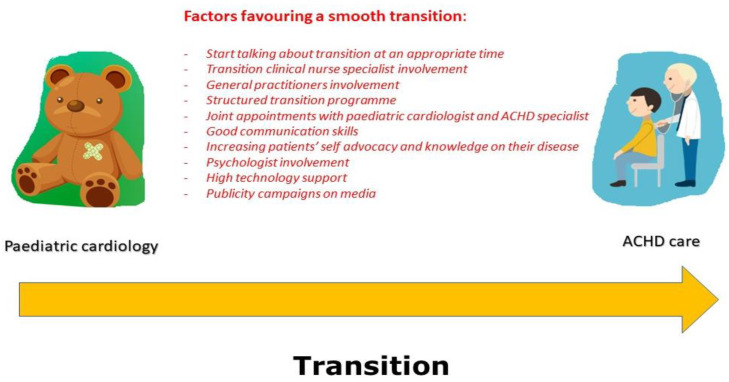
Strategies for a smooth transition from paediatric to adult CHD services.

What is sure is that the CHD population is living longer, growing in terms of number, and represents a vulnerable group. Though the problem is underestimated around the world, with some exceptions, implementing and refining protective measures aimed at reducing the loss of follow-up and related consequences in terms of morbidity and mortality is a burden that cannot be ignored any longer. With a synchronised and multidisciplinary endeavour, ultimately, subjects suffering from CHD should be able to achieve the goal of a smooth transition to adult-oriented follow-up (Figure 3) [85,86].

## Figures and Tables

**Table 1:** CHD classifications on the basis of complexity.

Disease Complexity	
Simple	- Isolated small atrial septal defect - Isolated small ventricular septal defect - Mild isolated pulmonary stenosis - Previously ligated or occluded ductus arteriosus - Repaired secundum atrial septal defect or sinus venosus defect without significant residual shunt or chamber enlargement - Repaired ventricular septal defect without significant residual shunt or chamber enlargement
Moderate complexity	- Aorto-left ventricular fistula - Anomalous pulmonary venous drainage, partial or total - Anomalous coronary artery arising from the pulmonary artery - Anomalous aortic origin of a coronary artery from the opposite sinus - Atrio-ventricular septal defect (partial or complete, including primum atrial septal defect) - Congenital aortic valve disease - Congenital mitral valve disease - Aortic coarctation - Ebstein anomaly (mild-to-severe variations) - Infundibular right ventricular outflow tract obstruction - Ostium primum atrial septal defect - Moderate and large unrepaired secundum atrial septal defect - Moderate and large persistently patent ductus arteriosus - Pulmonary valve regurgitation (moderate or greater) - Pulmonary valve stenosis (moderate or greater) - Peripheral pulmonary stenosis - Sinus of Valsalva fistula/aneurysm - Sinus venosus defect - Subvalvar aortic stenosis (excluding hypertrophic cardiomyopathy) - Supravalvar aortic stenosis - Straddling atrioventricular valve - Repaired tetralogy of Fallot - Ventricular septal defect with associated abnormality and/or moderate or greater shunt-
High complexity	- Cyanotic congenital heart defect (unrepaired or palliated, all forms) - Double-outlet ventricle - Fontan procedure - Interrupted aortic arch - Mitral atresia - Single ventricle (including double inlet left ventricle, tricuspid atresia, hypoplastic left heart, any other anatomic abnormality with a functionally single ventricle)
	- Cyanotic congenital heart defect (unrepaired or palliated, all forms) - Double-outlet ventricle - Fontan procedure - Interrupted aortic arch - Mitral atresia - Single ventricle (including double inlet left ventricle, tricuspid atresia, hypoplastic left heart, any other anatomic abnormality with a functionally single ventricle)
	- Pulmonary atresia (all forms) - Transposition of great vessels (classic or d-TGA; CCTGA or l-TGA) - Truncus arteriosus - Other abnormalities of atrioventricular and ventriculoarterial connection (i.e., crisscross heart, isomerism, heterotaxy syndromes, ventricular inversion)

## Data Availability

Not applicable.

## Supplementary Materials

The following supporting information can be downloaded at: https://www.mdpi.com/article/10.3390/children10030423/s1, PRISMA statement [87].
