# Supplementary material for: Strategies to Aid Successful Transition of Adolescents with Congenital Heart Disease: A Systematic Review
Source: Children (Basel). 2023 Feb 22;10(3):423. doi: 10.3390/children10030423 (PMC10047586; doi:10.3390/children10030423)
Supplement: Supplementary file 1 [file children-10-00423-s001.zip › children-2215319-supplementary.pdf]

PRISMA 2020 flow diagram for new systematic reviews which included searches of databases and registers only

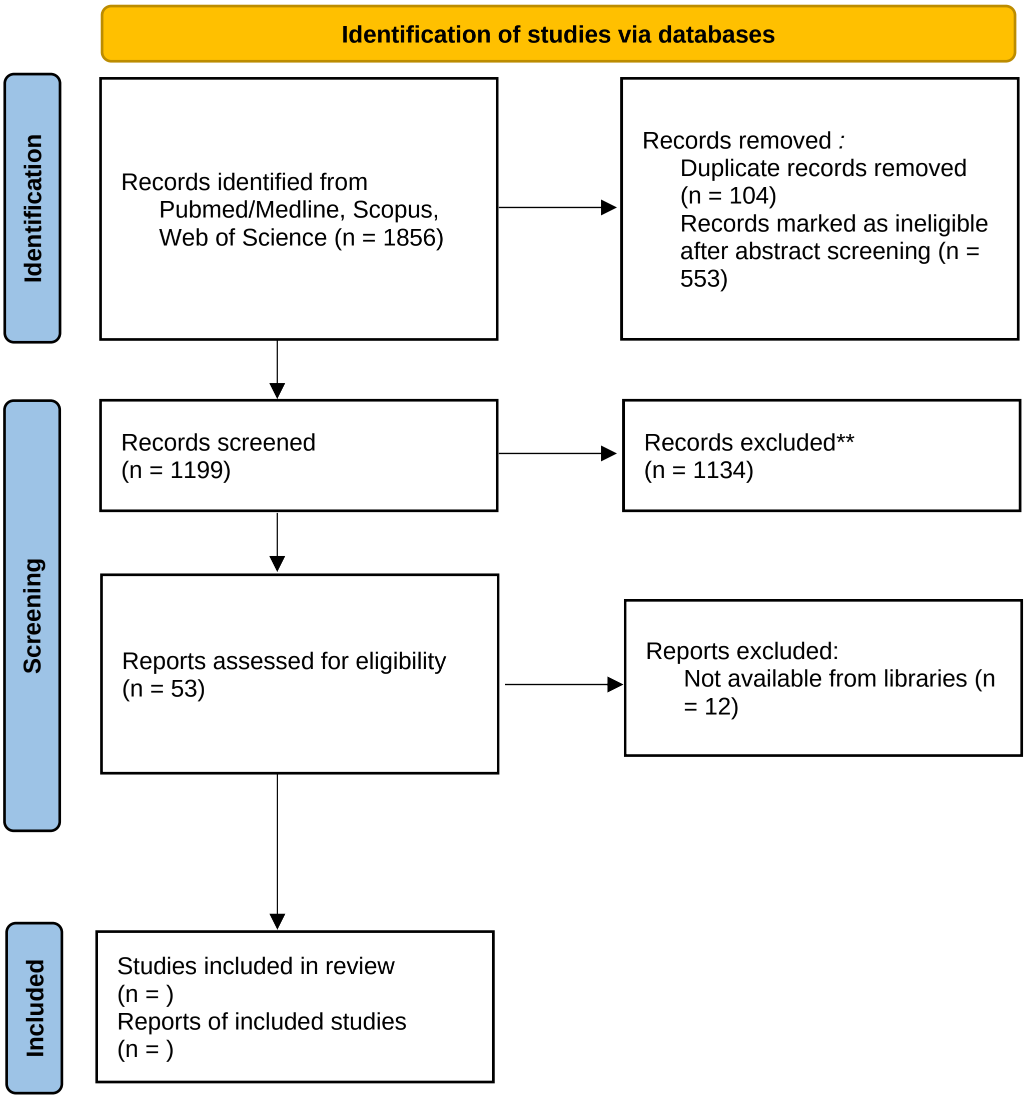

From: Page MJ, McKenzie JE, Bossuyt PM, Boutron I, Hoffmann TC, Mulrow CD, et al. The PRISMA 2020 statement: an updated guideline for reporting systematic reviews. BMJ 2021;372:n71. doi: 10.1136/bmj.n71
